# Association between triglyceride glucose-waist height ratio and stroke: a population-based study

**DOI:** 10.3389/fendo.2025.1510493

**Published:** 2025-03-12

**Authors:** Fangyuan Xu, Xingxing Su, Fan Dai, Yu Ye, Peijia Hu, Hongliang Cheng

**Affiliations:** ^1^ The First Clinical Medical School, Anhui University of Chinese Medicine, Hefei, China; ^2^ The Second Affiliated Hospital of Anhui University of Chinese Medicine, Hefei, China

**Keywords:** stroke, TyG-WHtR, NHANES, cross-sectional study, insulin resistance

## Abstract

**Background:**

Stroke poses a substantial threat to global public health. The triglyceride glucose-waist height ratio (TyG-WHtR), which incorporates the TyG metric with obesity-related WHtR, has demonstrated superior diagnostic and predictive value compared to the TyG index alone. Nevertheless, there is still a lack of in-depth exploration into the relationship between TyG-WHtR and stroke. This study seeks to address this gap by extracting information from the National Health and Nutrition Examination Survey (NHANES) to elucidate the potential association between TyG-WHtR levels and stroke.

**Methods:**

This study included 8,757 individuals from four research cycles conducted between 2011 and 2018. To examine the potential relationship between TyG-WHtR and stroke, we conducted multivariable logistic regression analysis. In addition, smooth curve fitting was applied to display the nonlinear association. Subgroup analyses and sensitivity analyses contributed to examining the robustness and consistency of the relationship between TyG-WHtR and stroke. The receiver operating characteristic (ROC) curves were employed to evaluate the diagnostic capability of TyG-WHtR and TyG.

**Results:**

After adjusting for relevant covariates, a positive association between TyG-WHtR levels and stroke occurrence was observed (OR: 1.26, 95% CI: 1.02-1.55). Specifically, each unit increase in TyG-WHtR was associated with a 26% higher likelihood of stroke. The findings of sensitivity analysis further demonstrated the stability of this positive relationship. Subgroup analysis revealed that this association was significant among participants who did not engage in moderate exercise and those without coronary heart disease or angina pectoris. ROC analysis demonstrated that TyG-WHtR exhibited superior predictive value compared to TyG.

**Conclusion:**

This study identified an association between elevated TyG-WHtR levels and an increased prevalence of stroke, suggesting that TyG-WHtR may serve as a valuable predictive tool for stroke risk, with potential implications for clinical prevention and early intervention.

## 1 Introduction

Stroke is a vital cause of long-term disability and mortality, representing a substantial threat to global public health (1). It is classified into ischemic and hemorrhagic types, with ischemic stroke (IS) being more prevalent, accounting for approximately 87% of all stroke occurrences worldwide (2). IS is primarily caused by acute arterial occlusion. Its etiologies include thromboembolism from large artery atherosclerosis, cardioembolism, small vessel disease, other identified causes such as hypercoagulable states and arterial dissection, and undetermined factors (3, 4). Hemorrhagic stroke, resulting from cerebral vascular injury or rupture, comprises two subtypes: intracerebral and subarachnoid hemorrhage. With an aging population, the incidence of stroke may constantly rise. According to 2017 data from the European Union, there were 1.12 million stroke events, 9.53 million stroke survivors, and 460,000 stroke-related deaths. By 2047, stroke events are projected to increase by 3%, while epidemic cases are expected to rise by 27%. However, stroke-related deaths and disability-adjusted life years are forecasted to decrease by 17% and 33%, respectively (5). The treatment and rehabilitation of stroke impose substantial economic burdens on both society and patients. In 2017, global medical costs related to stroke were estimated at $315 billion, with an additional $576 billion in lost income (6). These figures underscore the critical importance of early detection, intervention, and management to mitigate stroke progression.

Insulin resistance (IR) is characterized by weakened physiological function of insulin, wherein normal insulin levels fail to elicit an adequate physiological response (7). IR impairs endothelial function, enhances platelet aggregation, and promotes thrombosis, contributing to the development of IS. Moreover, it disrupts lipid metabolism, accelerating the formation of atherosclerosis and plaque (8, 9). Previous investigations have identified IR as a risk factor for stroke onset and progression, and it was related to poor functional prognosis following acute IS (7, 10, 11). Although the hyperinsulinemic-euglycemic clamp remains the gold standard for assessing IR, its widespread use is constrained by its time-consuming, labor-intensive, and costly nature (12). The triglyceride glucose (TyG), a reliable and cost-effective marker of IR, has been associated with higher stroke incidence in individuals without prior stroke history (13). A meta-analysis further established a significant link between the TyG indicator and the risk of stroke recurrence and mortality (14). The TyG-waist height ratio (WHtR), which integrates the TyG index with the obesity-related WHtR, has demonstrated superior diagnostic efficiency and predictive power for cardiovascular disease mortality compared to TyG (15). In conclusion, monitoring changes in these markers may aid in assessing metabolic function, potentially facilitating stroke prevention and improving prognosis.

Previous studies have revealed a positive correlation between TyG and TyG-body mass index (BMI) levels and stroke prevalence (16, 17). While these studies provide valuable insights, a noticeable gap remains in our understanding of the correlation between changes in TyG-WHtR and stroke occurrence. Therefore, this research seeks to elucidate the relationship between TyG-WHtR and stroke prevalence by analyzing relevant clinical data from NHANES.

## 2 Materials and methods

### 2.1 Study population

NHANES applies a stratified multistage probability sampling approach to investigate the health and nutritional status of the U.S. population. The survey is conducted every two years as a cycle. Data collection for NHANES research is approved and authorized by the Ethics Review Committee of the National Center for Health Statistics. Before the investigation began, each participant completed a written consent form. The survey collects a range of data, encompassing demographic data, physical examination results, dietary intake, laboratory findings, and responses to questionnaire surveys.

**Figure 1:**
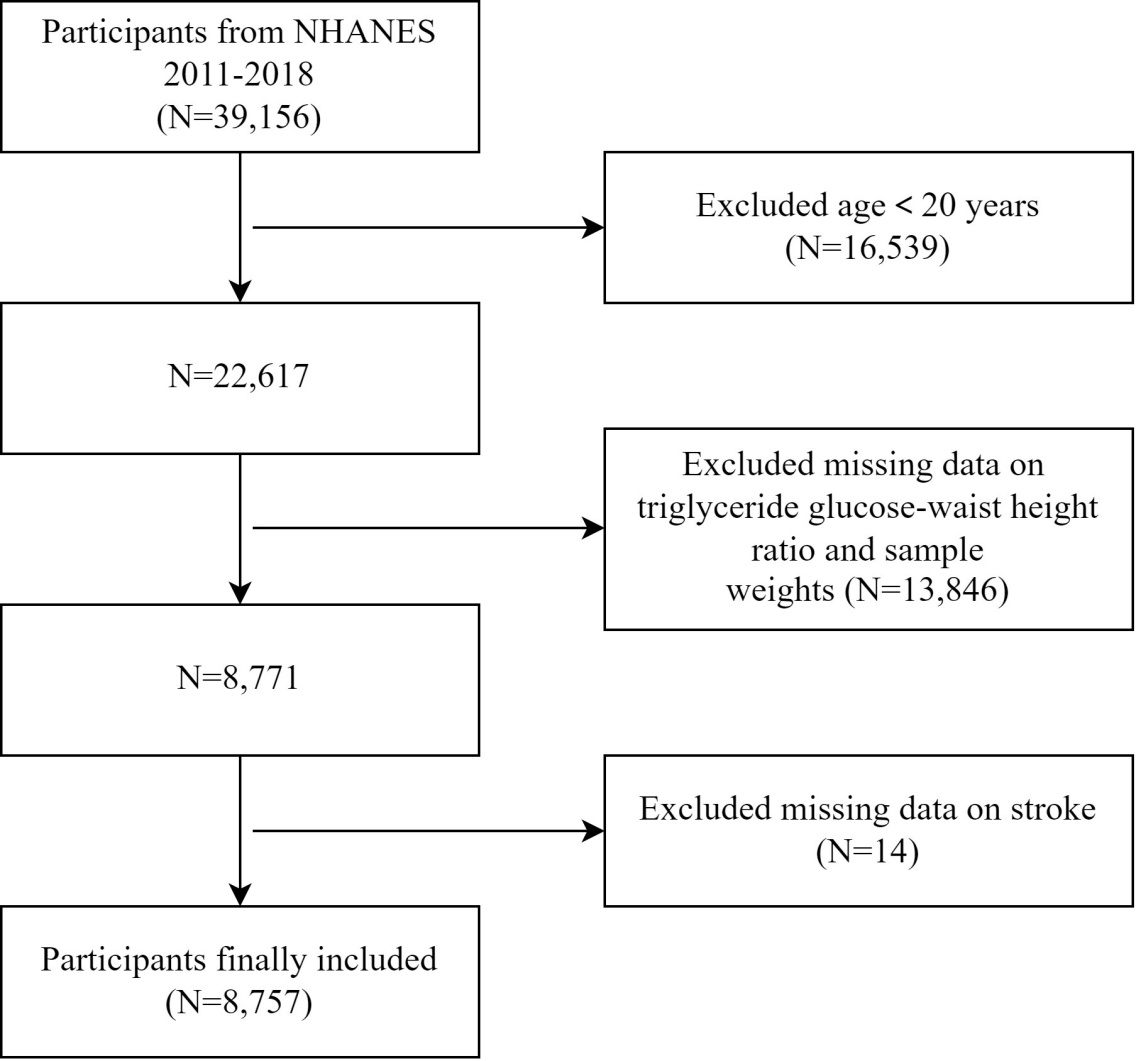
The flow diagram of participant selection from NHANES 2011 to 2018.

This cross-sectional study initially identified 39,156 participants from four consecutive NHANES cycles between 2011 and 2018. After data cleaning and sorting, we excluded 16,539 individuals under 20 years old, 11,502 with missing glucose levels, triglyceride levels, and sample weights, 2,316 with missing weight circumference (WC) data, 28 with missing height data, and 14 with missing stroke data. Ultimately, 8,757 individuals were selected for the subsequent analysis. A detailed flowchart outlining the data filtering process is presented in **Figure 1**.

### 2.2 Definition of stroke

Stroke occurrence was defined as the outcome variable, based on responses to a questionnaire survey administered by trained interviewers. Individuals were asked the following predetermined question: “Have doctors or other health professionals ever informed you that you had a stroke?” to determine whether they had experienced a stroke event. This self-reported method has been extensively utilized in prior studies investigating the association between stroke prevalence and various indices (18–20). Although the NHANES database does not distinguish between stroke subtypes, IS accounts for the majority of overall stroke cases, and IR is particularly related to IS occurrence (9). Consequently, this study primarily focuses on exploring the correlation between TyG-WHtR levels and IS prevalence.

### 2.3 Calculation of TyG-WHtR

The exposure variable in this study was TyG-WHtR. The following formulas were applied to calculate the relevant indicators: TyG = ln [fasting triglyceride (mg/dL) × fasting glucose (mg/dL)/2], WHtR= Waist circumference (cm)/Height (cm), while TyG-WHtR= TyG × WHtR.

### 2.4 Definition of covariables

By referring to previous similar studies (20–23), the covariates selected for this study include sex, age, race, marital status, educational level, average alcohol consumption, smoking status, BMI, total cholesterol (TC) level, cardiovascular diseases (coronary heart disease, heart failure, angina pectoris, and heart attack), diabetes status, hypertension status, and participation in moderate recreational activities. Education level was divided into three categories, using high school as the dividing point. Marital status was classified as either married or with a partner, and single. Participants who had smoked over 100 cigarettes in their whole life were defined as smokers. Alcohol consumption was evaluated based on average daily intake over the past 12 months. Comorbidities including hypertension, diabetes, and cardiovascular diseases (CVD) were determined through standardized questionnaire surveys. BMI was measured during physical examinations, and TC (mg/dL) was obtained from laboratory tests. Employing BMI thresholds of 25 and 30 kg/m^2^, participants were classified into three categories: normal weight, overweight, and obese.

### 2.5 Statistical analysis

This study explored participant characteristics based on distinct groups classified by TyG-WHtR levels and stroke status. Due to the complex sampling design of the NHANES survey, appropriate sample weights were applied in the subsequent analysis. Weighted categorical variables were expressed as percentages with 95% confidence intervals (CI), while non-normally distributed continuous variables were reported as medians with interquartile ranges. The chi-square test or the nonparametric Kruskal-Wallis test was utilized to compare data across groups with different TyG-WHtR levels and between stroke and non-stroke groups. Missing covariate data were addressed using multiple imputation, which generated five imputed datasets. The results were then combined for analysis. To identify the potential relationship between TyG-WHtR levels and stroke occurrence, this study performed multivariable logistic regression analysis. Three distinct models were used to investigate this relationship: Model 1, which included no adjustments for variables; Model 2, which was partially adjusted for sex, age, race, and BMI; and Model 3, which was fully adjusted, incorporating additional covariates such as marital status, educational level, average alcohol consumption, BMI, TC, CVD, diabetes status, hypertension status, smoking status, and engagement in moderate recreational activities. Subgroup analyses were implemented to clarify the stability and consistency of the relationship between TyG-WHtR levels and stroke occurrence across different populations, stratified by specific variables. When interaction tests were not statistically significant, the association was considered consistent across subgroups. A generalized additive model was then employed to perform smooth curve fitting, examining potential nonlinear associations between TyG-WHtR and stroke occurrence. Sensitivity analysis assessed the robustness of the correlation by excluding missing covariates. Finally, the receiver operating characteristic (ROC) curves were applied to examine diagnostic capability, and the area under the curve (AUC) was calculated to explain the predictive performance of TyG and TyG-WHtR for stroke. All statistical analyses were conducted using R 4.1.3 and Empower software.

## 3 Results

### 3.1 Baseline characteristics of participants

This study included 8,757 individuals, of whom 48.52% were male and 51.48% were female. Participants diagnosed with stroke comprised 2.84% of the total population. **Table 1** displays the clinical characteristics of individuals categorized by quartiles of TyG-WHtR levels. Statistical differences were observed across quartiles for variables such as sex, age, race, smoking, marital status, educational level, BMI categories, TC, CVD, diabetes status, hypertension status, and participation in moderate recreational activities. In comparison to individuals in Q1, those in Q4 were older, had higher TC levels, and were more likely to be female, non-Hispanic White, married or partnered, smokers, and classified as obese. What’s more, a higher percentage of individuals in Q4 had diabetes, hypertension, CVD, and a history of stroke. Conversely, fewer participants in Q4 had an education level above high school or engaged in moderate recreational activities. **Table 2** summarizes the clinical information of individuals stratified by stroke diagnosis. Notably, stroke patients were older, had higher TyG-WHtR levels, and lower TC levels compared to the non-stroke population. They are also more prone to suffering from hypertension, diabetes, and four types of CVD. Among stroke patients, a greater proportion were smokers, had higher educational attainment, and did not participate in moderate physical activities.

**Table 1:** The characteristics of participants by TyG-WHtR level among U.S. adults.

Characteristics	Q1	Q2	Q3	Q4	*P*-value
Age (years)	36.00 (26.00, 51.00)	49.00 (35.00, 61.00)	50.00 (38.00, 63.00)	54.00 (40.00, 64.00)	<0.001
Sex (%)					<0.001
Male	47.10 (44.31, 49.90)	53.20 (50.25, 56.12)	51.98 (49.29, 54.66)	41.57 (38.80, 44.39)	
Female	52.90 (50.10, 55.69)	46.80 (43.88, 49.75)	48.02 (45.34, 50.71)	58.43 (55.61, 61.20)	
Race (%)					<0.001
Non-Hispanic White	63.60 (59.16, 67.83)	65.71 (61.83, 69.39)	63.17 (59.34, 66.83)	67.25 (62.88, 71.33)	
Non-Hispanic Black	13.72 (11.34, 16.50)	10.48 (8.58, 12.73)	10.17 (8.45, 12.19)	10.08 (8.07, 12.51)	
Mexican American	5.05 (3.84, 6.61)	8.20 (6.54, 10.25)	10.89 (8.75, 13.49)	11.11 (8.64, 14.18)	
Other races	17.63 (15.08, 20.50)	15.61 (13.37, 18.15)	15.77 (13.64, 18.18)	11.57 (9.94, 13.42)	
Education level (%)					<0.001
< high school	11.61 (9.35, 14.33)	13.46 (11.55, 15.62)	16.65 (14.36, 19.23)	18.29 (16.42, 20.33)	
High school	18.74 (16.11, 21.70)	22.41 (19.33, 25.82)	25.06 (22.20, 28.15)	23.82 (21.18, 26.67)	
> high school	69.65 (65.17, 73.77)	64.13 (60.40, 67.70)	58.29 (54.27, 62.20)	57.89 (54.61, 61.11)	
Marital status (%)					<0.001
Married or with partner	58.39 (55.06, 61.64)	66.72 (63.28, 69.98)	68.34 (65.16, 71.35)	61.83 (58.32, 65.21)	
Single	41.61 (38.36, 44.94)	33.28 (30.02, 36.72)	31.66 (28.65, 34.84)	38.17 (34.79, 41.68)	
Smoking (%)					<0.001
Yes	38.69 (35.23, 42.26)	43.47 (40.40, 46.60)	45.99 (42.98, 49.02)	47.77 (45.15, 50.40)	
No	61.31 (57.74, 64.77)	56.53 (53.40, 59.60)	54.01 (50.98, 57.02)	52.23 (49.60, 54.85)	
Diabetes (%)					<0.001
Yes	2.02 (1.50, 2.73)	7.22 (5.84, 8.89)	12.90 (10.70, 15.47)	28.92 (26.49, 31.48)	
No	97.98 (97.27, 98.50)	92.78 (91.11, 94.16)	87.10 (84.53, 89.30)	71.08 (68.52, 73.51)	
Hypertension (%)					<0.001
Yes	12.83 (10.84, 15.13)	30.05 (27.33, 32.92)	39.09 (36.43, 41.81)	51.83 (48.70, 54.94)	
No	87.17 (84.87, 89.16)	69.95 (67.08, 72.67)	60.91 (58.19, 63.57)	48.17 (45.06, 51.30)	
Stroke					0.002
Yes	1.63 (0.96, 2.76)	2.72 (1.92, 3.84)	2.83 (2.10, 3.80)	4.32 (3.36, 5.55)	
No	98.37 (97.24, 99.04)	97.28 (96.16, 98.08)	97.17 (96.20, 97.90)	95.68 (94.45, 96.64)	
Coronary heart disease (%)					<0.001
Yes	1.49 (0.88, 2.51)	3.50 (2.56, 4.77)	3.19 (2.31, 4.40)	5.80 (4.31, 7.76)	
No	98.51 (97.49, 99.12)	96.50 (95.23, 97.44)	96.81 (95.60, 97.69)	94.20 (92.24, 95.69)	
Heart attack (%)					<0.001
Yes	1.38 (0.80, 2.36)	3.26 (2.40, 4.41)	3.74 (2.88, 4.84)	5.10 (4.03, 6.44)	
No	98.62 (97.64, 99.20)	96.74 (95.59, 97.60)	96.26 (95.16, 97.12)	94.90 (93.56, 95.97)	
Angina pectoris (%)					<0.001
Yes	0.95 (0.48, 1.88)	1.75 (1.21, 2.52)	2.25 (1.52, 3.31)	3.86 (2.67, 5.55)	
No	99.05 (98.12, 99.52)	98.25 (97.48, 98.79)	97.75 (96.69, 98.48)	96.14 (94.45, 97.33)	
Heart failure (%)					<0.001
Yes	1.06 (0.60, 1.87)	1.69 (1.18, 2.42)	2.22 (1.60, 3.06)	5.09 (4.03,6.41)	
No	98.94 (98.13, 99.40)	98.31 (97.58, 98.82)	97.78 (96.94, 98.40)	94.91 (93.59, 95.97)	
Moderate recreational activities (%)					<0.001
Yes	54.76 (51.07, 58.39)	49.00 (45.12, 52.89)	43.45 (40.54, 46.41)	36.32 (33.56, 39.17)	
No	45.24 (41.61, 48.93)	51.00 (47.11, 54.88)	56.55 (53.59, 59.46)	63.68 (60.83, 66.44)	
BMI category (%)					<0.001
Normal weight (< 25 kg/m^2^)	79.26 (76.70, 81.61)	25.69 (22.90, 28.70)	4.62 (3.65, 5.84)	0.38 (0.18, 0.77)	
Overweight (25-30 kg/m^2^)	19.86 (17.61, 22.31)	58.30 (55.46, 61.10)	43.25 (40.64, 45.90)	8.61 (7.19, 10.28)	
Obese (≥30 kg/m^2^)	0.88 (0.55, 1.42)	16.00 (14.30, 17.87)	52.13 (49.18, 55.07)	91.01 (89.29, 92.48)	
Average alcohol intake	2.00 (1.00, 3.00)	1.00 (1.00, 2.00)	1.00 (1.00, 3.00)	1.00 (1.00, 2.00)	0.836
TC (mg/dL)	176.00 (155.00, 201.00)	189.00 (164.00, 215.00)	194.00 (167.00, 223.00)	192.00 (165.00, 220.00)	<0.001

Categorical variables: weighted percentages (95% CI), non-normally distributed continuous variables: weighted median (Q1, Q3). TyG-WHtR, triglyceride glucose-waist height ratio; BMI, body mass index; TC, total cholesterol; CI, confidence interval.

**Table 2:** The characteristics of participants classified by stroke status.

Characteristics	Non-Stroke	Stroke	*P*-value
Age (years)	46.00 (33.00, 60.00)	66.00 (57.00, 75.00)	<0.001
Sex (%)			0.124
Male	48.69 (47.53, 49.85)	42.58 (35.17, 50.35)	
Female	51.31 (50.15, 52.47)	57.42 (49.65, 64.83)	
Race/ethnicity (%)			0.007
Non-Hispanic White	64.90 (61.25, 68.38)	65.13 (57.96, 71.67)	
Non-Hispanic Black	11.01 (9.21, 13.12)	16.42 (12.53, 21.23)	
Mexican American	8.82 (7.07, 10.96)	4.78 (3.11, 7.28)	
Other races	15.27 (13.59, 17.11)	13.67 (9.30, 19.64)	
Education level (%)			<0.001
< high school	14.56 (12.88, 16.42)	25.95 (20.62, 32.10)	
High school	22.23 (20.50, 24.06)	28.51 (22.12, 35.90)	
> high school	63.21 (60.38, 65.94)	45.54 (37.64, 53.67)	
Marital status (%)			0.633
Married or with partner	63.77 (61.67, 65.83)	62.24 (55.63, 68.43)	
Single	36.23 (34.17, 38.33)	37.76 (31.57, 44.37)	
Smoking (%)			<0.001
Yes	43.35 (41.45, 45.26)	60.39 (54.14, 66.31)	
No	56.65 (54.74, 58.55)	39.61 (33.69, 45.86)	
Diabetes (%)			<0.001
Yes	11.84 (10.88, 12.86)	30.58 (24.94, 36.86)	
No	88.16 (87.14, 89.12)	69.42 (63.14, 75.06)	
Hypertension (%)			<0.001
Yes	31.82 (30.08, 33.61)	67.44 (59.58, 74.43)	
No	68.18 (66.39, 69.92)	32.56 (25.57, 40.42)	
Coronary heart disease (%)			<0.001
Yes	3.09 (2.53, 3.79)	15.20 (10.66, 21.22)	
No	96.91 (96.21, 97.47)	84.80 (78.78, 89.34)	
Heart attack (%)			<0.001
Yes	2.92 (2.41, 3.54)	16.77 (11.24, 24.28)	
No	97.08 (96.46, 97.59)	83.23 (75.72, 88.76)	
Angina pectoris (%)			<0.001
Yes	1.89 (1.52, 2.36)	11.21 (7.30, 16.82)	
No	98.11 (97.64, 98.48)	88.79 (83.18, 92.70)	
Heart failure (%)			<0.001
Yes	2.01 (1.70, 2.37)	17.96 (13.38, 23.68)	
No	97.99 (97.63, 98.30)	82.04 (76.32, 86.62)	
Moderate recreational activities (%)			<0.001
Yes	46.56 (44.43, 48.70)	33.05 (25.95, 41.02)	
No	53.44 (51.30, 55.57)	66.95 (58.98, 74.05)	
Average alcohol (d)	1.00 (1.00, 2.00)	1.00 (1.00, 2.00)	<0.001
BMI category (%)			0.536
Normal weight (< 25 kg/m^2^)	28.89 (27.14, 30.70)	26.87 (20.83, 33.90)	
Overweight (25-30 kg/m^2^)	32.69 (31.50, 33.90)	30.51 (24.11, 37.76)	
Obese (≥30 kg/m^2^)	38.42 (36.64, 40.24)	42.62 (34.79, 50.85)	
TC (mg/dL)	187.00 (162.00, 214.00)	177.00 (153.00, 206.00)	0.004
TyG-WHtR	5.01 (4.32, 5.76)	5.42 (4.65, 6.21)	<0.001

Categorical variables: weighted percentages (95% CI), non-normally distributed continuous variables: weighted median (Q1, Q3). TyG-WHtR, triglyceride glucose-waist height ratio; BMI, body mass index; TC, total cholesterol; CI, confidence interval.

### 3.2 Association between TyG-WHtR and stroke

**Figure 2:**
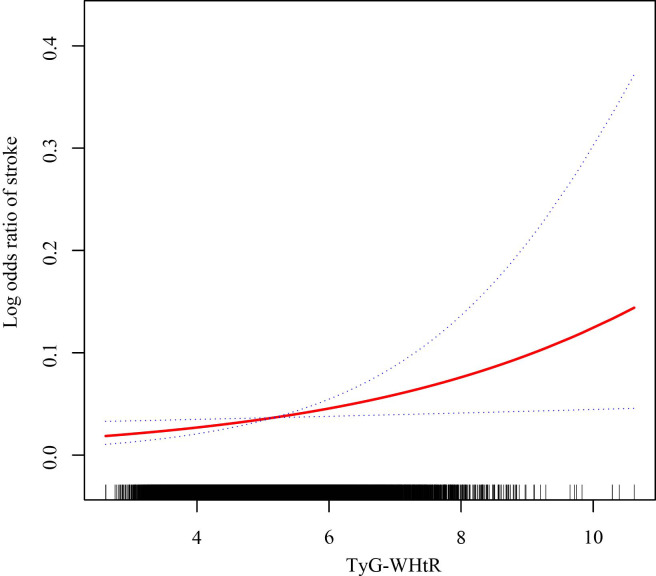
The association between the TyG-WHtR level and stroke prevalence. The solid red line manifests the smooth curve fit between variables. The blue dotted lines indicate the 95% CI from the fit.

The relationship between TyG-WHtR levels and stroke occurrence was evaluated using multivariable logistic regression models. As illustrated in **Table 3**, a significant positive association was identified in the unadjusted Model 1. This association remained significant in the partially-adjusted Model 2 (OR: 1.61, 95% CI: 1.34-1.95) and the fully-adjusted Model 3 (OR: 1.26, 95% CI: 1.02-1.55). Based on the results from Model 3, each unit additional TyG-WHtR value was associated with a 26% rise in the odds of stroke. Subsequently, we conducted a more in-depth analysis by categorizing the TyG-WHtR index into quartiles. In Model 2, compared to Quartile 1, participants in Q4 had a significantly higher probability of experiencing a stroke (OR: 3.20, 95% CI: 1.43-7.14). The trend test demonstrated statistical significance in Models 1 and 2 only. As depicted in **Figure 2**, the smooth curve fitting analysis further verified the positive correlation between TyG-WHtR levels and stroke occurrence.

**Table 3:** The association between the TyG-WHtR and stroke prevalence.

Exposure	Model 1	Model 2	Model 3
	[OR (95% CI)] *P*-value	[OR (95% CI)] *P*-value	[OR (95% CI)] *P*-value
TyG-WHtR	1.40 (1.23, 1.60) <0.001	1.61 (1.34, 1.95) <0.001	1.26 (1.02, 1.55) 0.037
Category TyG-WHtR (quartile)			
Quartile 1	Reference	Reference	Reference
Quartile 2	1.68 (0.87, 3.27) 0.129	1.60 (0.78, 3.27) 0.207	1.25 (0.60, 2.63) 0.553
Quartile 3	1.76 (1.03, 2.99) 0.043	1.93 (0.97, 3.86) 0.066	1.22 (0.61, 2.44) 0.569
Quartile 4	2.72 (1.47, 5.06) 0.002	3.20 (1.43, 7.14) 0.006	1.46 (0.65, 3.32) 0.368
*P* for trend	<0.001	0.005	0.393

Model 1: No covariates were adjusted. Model 2: Sex, age, race, and BMI were adjusted. Model 3: Sex, age, race, marital status, education level, average alcohol intake, smoking, BMI, TC, coronary heart disease, heart attack, angina pectoris, heart failure, diabetes status, hypertension status, and moderate recreational activities were adjusted. TyG-WHtR, triglyceride glucose-waist height ratio; OR, odds ratio; CI, confidence interval.

### 3.3 Subgroup analysis

To examine the consistency of the correlation between TyG-WHtR levels and stroke prevalence across different populations, subgroup analyses and interaction tests were conducted according to the stratification factors encompassing sex, age, race, hypertension, coronary heart disease (CHD), diabetes, angina pectoris, heart failure, smoking status, moderate recreational activities, and BMI. As shown in **Table 4**, significant interactions were observed for CHD, angina pectoris, and exercise (*P* for interaction < 0.05). In the subgroups of CHD and angina pectoris, a significant correlation was found among participants without CHD (OR: 1.35; 95% CI: 1.09-1.67) or angina pectoris (OR: 1.32; 95% CI: 1.09-1.61). Regarding the stratified factor of exercise, a significant positive association between TyG-WHtR and stroke was detected only in individuals who did not engage in moderate recreational exercise (OR: 1.40; 95%CI: 1.13-1.73). What’s more, the association between TyG-WHtR and stroke remained stable across populations stratified by sex, race, age, diabetes, smoking status, hypertension, heart failure, and BMI categories (all *P* for interaction > 0.05).

**Table 4:** Subgroup analysis of the association between the TyG-WHtR and stroke.

Subgroup	TyG-WHtR [OR (95%CI)] *P*-value	*P* for interaction
Sex		0.414
Male	1.36 (1.03, 1.80) 0.038	
Female	1.21 (0.96, 1.51) 0.116	
Age		0.252
< 60 years	1.36 (1.11, 1.66) 0.005	
≥ 60 years	1.16 (0.86, 1.54) 0.335	
Race		0.506
Non-Hispanic White	1.21 (0.93, 1.57) 0.170	
Non-Hispanic Black	1.37 (1.10, 1.71) 0.008	
Mexican American	1.19 (0.81, 1.76) 0.374	
Other races	1.45 (1.10, 1.90) 0.011	
Hypertension		0.101
Yes	1.16 (0.91, 1.46) 0.234	
No	1.51 (1.12, 2.04) 0.009	
Diabetes		0.116
Yes	1.45 (1.09, 1.93) 0.014	
No	1.13 (0.88, 1.43) 0.342	
Coronary heart disease		0.002
Yes	0.67 (0.44, 1.01) 0.066	
No	1.35 (1.09, 1.67) 0.010	
Angina pectoris		0.021
Yes	0.66 (0.35, 1.24) 0.204	
No	1.32 (1.09, 1.61) 0.008	
Heart failure		0.083
Yes	0.95 (0.65, 1.39) 0.783	
No	1.33 (1.08, 1.63) 0.010	
Smoking		0.438
Yes	1.20 (0.92, 1.56) 0.191	
No	1.35 (1.06, 1.71) 0.019	
Moderate recreational activities		0.003
Yes	0.90 (0.66, 1.22) 0.483	
No	1.40 (1.13, 1.73) 0.004	
BMI category		0.235
Normal weight	1.50 (0.86, 2.61) 0.162	
Overweight	0.89 (0.56, 1.40) 0.611	
Obesity	1.32 (1.03, 1.70) 0.033	

Sex, age, race, marital status, education level, average alcohol intake, smoking, BMI, TC, coronary heart disease, heart attack, angina pectoris, heart failure, diabetes status, hypertension status, and moderate recreational activities were adjusted. TyG-WHtR, triglyceride glucose-waist height ratio; BMI, body mass index; TC, total cholesterol; OR, odds ratio; CI, confidence interval.

### 3.4 ROC curves analysis

**Figure 3:**
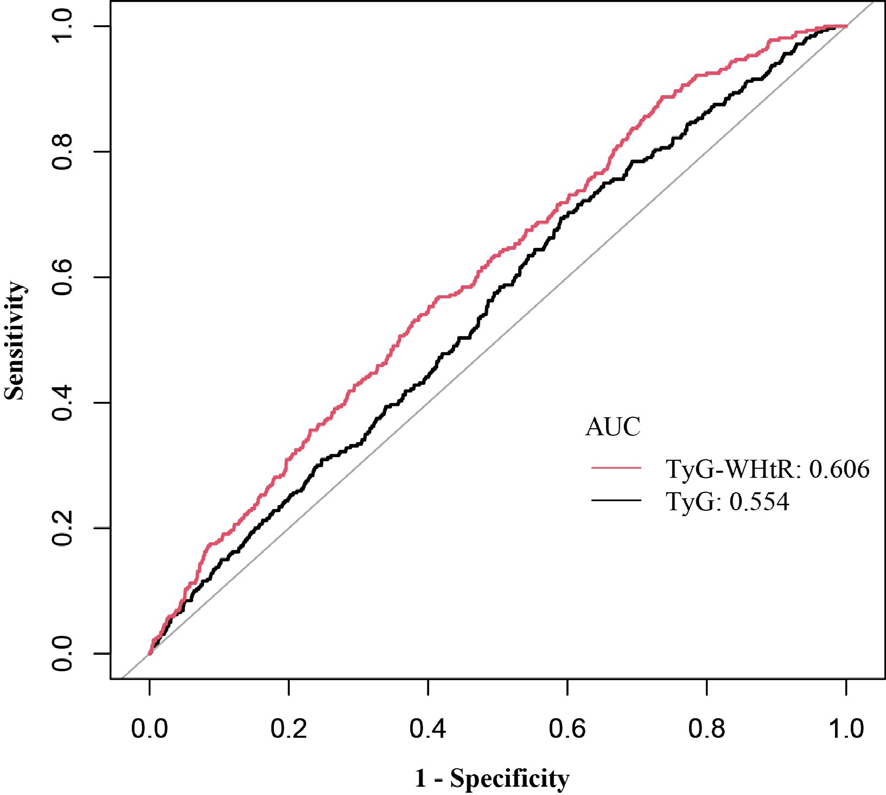
ROC curves and AUC values of TyG and TyG-WHtR in diagnosing stroke. ROC, receiver operating characteristic; AUC, area under the curve; TyG, triglyceride glucose; TyG-WHtR, triglyceride glucose-waist height ratio.

ROC curves were applied to examine the diagnostic performance of TyG and TyG-WHtR for stroke, with AUC manifesting predictive accuracy. As depicted in **Table 5**, **Figure 3**, TyG-WHtR exhibited superior predictive capability (AUC: 0.606, 95% CI: 0.58-0.64) compared to TyG (AUC: 0.554, 95% CI: 0.52-0.59). Additionally, the optimal threshold for predicting stroke using TyG-WHtR is 5.29.

**Table 5:** Comparison of TyG and TyG-WHtR for predicting stroke.

Test	ROC area (AUC)	95%CI lower	95%CI upper	Best threshold	Specificity	Sensitivity
TyG	0.554	0.52	0.59	8.38	0.41	0.69
TyG-WHtR	0.606	0.58	0.64	5.29	0.59	0.57

ROC, receiver operating characteristic; AUC, area under the curve; TyG, triglyceride glucose; TyG-WHtR, triglyceride glucose-waist height ratio; CI, confidence interval.

### 3.5 Sensitivity analysis

The results of the multivariable regression analysis, conducted after removing missing covariates, further confirm the consistent and robust positive correlation between TyG-WHtR level and stroke occurrence (OR: 1.32; 95% CI: 1.06-1.65) (**Supplementary Table S1**).

## 4 Discussion

This study is the first to explore the association between TyG-WHtR and stroke occurrence among Americans. A total of 8,757 representative participants were included, and a significant positive correlation of TyG-WHtR levels and stroke prevalence was observed. This association remained robust after making adjustments for relevant covariables. Specifically, each unit increase of TyG-WHtR was associated with a 26% higher likelihood of stroke. Subgroup analyses further indicated that stratification of CHD, angina pectoris, and moderate recreational exercise may influence this relationship. Consistent with these findings, a meta-analysis revealed that elevated TyG levels were independently linked to an increased stroke incidence in people without preexisting atherosclerotic cardiovascular disease (24). For individuals with CHD or angina pectoris, the use of lipid-lowering and antiplatelet medications may attenuate the association between TyG-WHtR and stroke prevalence. Physical exercise induces beneficial changes in lipid metabolism and enhances insulin sensitivity through various adaptations in glucose transport and metabolism (25). Therefore, physical activity may mitigate the impact of TyG-WHtR-related metabolic disorders on stroke. ROC analysis further demonstrated that TyG-WHtR had superior predictive value compared to TyG. Therefore, TyG-WHtR may be considered a valuable and cost-effective marker for stroke prevention and early identification, particularly in individuals who are not engaged in moderate physical exercise and those without CHD or angina pectoris. Special attention should be given to these populations when managing stroke risk and evaluating the role of TyG-WHtR in stroke prediction.

IR can aggravate atherosclerosis and contribute to hemodynamic disorders, which are closely related to the occurrence, early neurological deterioration, recurrence, and poor prognosis of IS (10, 26, 27). TyG is an effective and simple alternative marker for identifying IR. Evidence suggests that TyG has been broadly employed in research on CVD, type 2 diabetes mellitus, depression, chest pain, and stroke (15, 28–30). A study involving 10,132 individuals from American communities found that higher TyG levels were linked to an elevated risk of stroke and IS (31). Previous investigations have further demonstrated a link between IR and CVD in non-hypertensive populations (32). Moreover, a large-scale Chinese study with an 11-year follow-up revealed the predictive value of the TyG indicator for stroke and IS, though it did not exhibit a consistent predictive effect for ICH (33).

Metabolic syndrome (MetS) is a pathological condition featured as metabolic abnormalities, encompassing IR, hypertension, atherogenic dyslipidemia, and abdominal obesity (34, 35). Obesity-related metrics are often combined with the TyG index to evaluate MetS. A national cohort study reported that TyG-BMI, TyG-WC, and TyG-WHtR have superior predictive effects for MetS compared to simple obesity- or lipid-related indicators, as well as TyG alone. A Chinese cohort study of 4,583 individuals showed that TyG-BMI levels were linked to stroke risk among individuals aged 45 and older (36). Additionally, a study revealed that TyG and related metrics were positively related to atherosclerotic cardiovascular disease (37). WHtR is an easily accessible and useful metric for evaluating abdominal obesity, with previous studies highlighting its predictive role for stroke risk and its influence on post-stroke functional recovery (38–40). However, the exploration of the relationship between TyG-WHtR level and the probability of suffering a stroke remains insufficient. Therefore, the current study fills the insufficiency and points out their positive correlation. Our research findings emphasize the importance of multidimensional stroke prevention in both clinical practice and public health. Obesity serves as a key risk factor for stroke, and effective weight management may be essential for reducing stroke incidence and slowing disease progression. A meta-analysis illustrated that bariatric surgery decreased stroke incidence, especially IS (41). Moreover, reducing obesity and improving metabolism through physical exercise, a healthy diet, and lifestyle modifications may help alleviate the substantial burden of stroke (42).

The potential mechanism linking TyG-WHtR to stroke requires further elucidation. Atherosclerosis, a long-term inflammatory disorder, is closely related to stroke occurrence (43). This process is influenced by multiple factors such as IR, hyperglycemia, inflammation, and dyslipidemia (44, 45). Abnormal insulin signaling in endothelial cells (ECs), vascular smooth muscle cells (VSMCs), and macrophages accelerates the formation and development of arteriosclerosis. In ECs, downregulation of the insulin receptor-Akt1 signaling pathway leads to endothelial dysfunction and an increase in the entry of inflammatory cells into plaques (46). In addition, IR promotes the proliferation of VSMCs, thereby accelerating plaque growth (47). In hyperinsulinemic and obese states, the insulin receptors of macrophages are downregulated, and intimal macrophages contribute to atherosclerosis by participating in inflammation and secreting protease and procoagulant factors (46, 48). Hyperglycemia results in excessive reactive oxygen species (ROS) production, induces pro-inflammatory responses, and promotes advanced glycation end-products formation through protein glycoxidation and glycation (44, 49). Furthermore, obesity promotes ectopic lipid deposition, induces lipotoxicity, releases excessive ROS, and triggers endoplasmic reticulum stress and inflammatory responses. These metabolic disturbances lead to the development of IR and chronic low-grade inflammatory condition, both of which are considered pivotal in the pathogenesis of stroke (50, 51).

This exploration has multiple strengths. First, the extensive sample size and inclusion of representative individuals enhance the robustness and generalizability of the findings. Second, stratifying TyG-WHtR into quartiles and categorizing participants by stroke status enabled a more effective assessment of the impact of TyG-WHtR levels on population characteristics and the distinct features of stroke patients. Third, comprehensive adjustments for potential confounders minimized bias and improved the reliability of the results. Finally, subgroup analyses revealed a significant correlation between TyG-WHtR levels and stroke in individuals who did not participate in moderate exercise and in those without CHD or angina pectoris, providing valuable insights for stroke prevention and intervention strategies.

Nevertheless, we acknowledge several limitations that should be considered when interpreting these findings. First, due to the constraints of a cross-sectional design, a causal link between TyG-WHtR and stroke cannot be established. Second, the reliance on self-reported comorbidities may introduce recall bias. Additionally, the study does not differentiate between stroke subtypes. Finally, although multiple confounding factors were considered, the potential impact of unaccounted variables cannot be excluded.

## 5 Conclusion

This study identified an association between elevated TyG-WHtR levels and increased stroke prevalence, underscoring the importance of blood sugar and lipid management in stroke prevention. These findings suggest that TyG-WHtR may function as a valuable predictive metric for stroke, offering potential guidance for clinical prevention and early intervention. However, prospective clinical trials are needed to further validate these results and provide valuable insights for clinical practice.

## Data Availability

The original contributions presented in the study are included in the article/**Supplementary Material**. Further inquiries can be directed to the corresponding authors.
