# Supplementary material for: Association between triglyceride glucose-waist height ratio and stroke: a population-based study
Source: Front Endocrinol (Lausanne). 2025 Mar 12;16:1510493. doi: 10.3389/fendo.2025.1510493 (PMC11936810; doi:10.3389/fendo.2025.1510493)
Supplement: Supplementary file 1 [file Table1.doc]

**TABLE S1 The association between the TyG-WHtR and stroke prevalence after excluding missing covariables.**

| **Exposure** | **Model 1** | **Model 2** | **Model 3** |
| --- | --- | --- | --- |
|  | **[OR (95% CI)] *P*-value** | **[OR (95% CI)] *P*-value** | **[OR (95% CI)] *P*-value** |
| TyG-WHtR | 1.29 (1.17, 1.42) <0.001 | 1.86 (1.54, 2.26) <0.001 | 1.32 (1.06, 1.65) 0.014 |
| Category TyG-WHtR (quartile) |  |  |  |
| Quartile 1 | Reference | Reference | Reference |
| Quartile 2 | 1.44 (1.00, 2.07) 0.047 | 1.37 (0.93, 2.02) 0.112 | 1.18 (0.79, 1.77) 0.418 |
| Quartile 3 | 1.77 (1.25, 2.50) 0.001 | 1.83 (1.20, 2.78) 0.005 | 1.36 (0.88, 2.12) 0.171 |
| Quartile 4 | 2.20 (1.57, 3.08) <0.001 | 2.92 (1.75, 4.87) <0.001 | 1.49 (0.86, 2.60) 0.159 |
| *P* for trend | <0.001 | <0.001 | 0.153 |

Model 1: No covariates were adjusted. Model 2: Sex, age, race, and BMI were adjusted. Model 3: Sex, age, race, marital status, education level, average alcohol intake, smoking, BMI, TC, coronary heart disease, heart attack, angina pectoris, heart failure, diabetes status, hypertension status, and moderate recreational activities were adjusted. TyG-WHtR, triglyceride glucose-waist height ratio; OR, odds ratio; CI, confidence interval.
